# Photodynamic Therapy in Non-Gastrointestinal Thoracic Malignancies

**DOI:** 10.3390/ijms17010135

**Published:** 2016-01-21

**Authors:** Biniam Kidane, Dhruvin Hirpara, Kazuhiro Yasufuku

**Affiliations:** 1 Division of Thoracic Surgery, University of Toronto, Toronto, ON M5G 2C4, Canada; b.kidane@mail.utoronto.ca; 2 Division of Thoracic Surgery, Toronto General Hospital, Toronto, ON M5G 2C4, Canada; 3 Faculty of Medicine, University of Toronto, Toronto, ON M5S 1A8, Canada; dhruvinh@gmail.com

**Keywords:** photodynamic therapy, non-small cell lung cancer, thoracic malignancy, mesothelioma, bronchial obstruction, airway obstruction, pleural metastasis

## Abstract

Photodynamic therapy has a role in the management of early and late thoracic malignancies. It can be used to facilitate minimally-invasive treatment of early endobronchial tumours and also to palliate obstructive and bleeding effects of advanced endobronchial tumours. Photodynamic therapy has been used as a means of downsizing tumours to allow for resection, as well as reducing the extent of resection necessary. It has also been used successfully for minimally-invasive management of local recurrences, which is especially valuable for patients who are not eligible for radiation therapy. Photodynamic therapy has also shown promising results in mesothelioma and pleural-based metastatic disease. As new generation photosensitizers are being developed and tested and methodological issues continue to be addressed, the role of photodynamic therapy in thoracic malignancies continues to evolve.

## 1. Introduction

Photodynamic therapy (PDT) has been used increasingly in the treatment of thoracic malignancies. PDT involves systemic treatment with a photosensitizing agent, which is selectively retained in higher concentrations in tumor cells than in surrounding tissue [1,2]. The agent is then activated at specific sites by direct application of light at a wavelength corresponding to the absorption band of the photosensitizing agent. In the presence of oxygen, this results in a photodynamic reaction, thereby exerting local cytotoxic effects [1]. These local cytotoxic effects include direct tumour cell death (both via apoptotic and necrotic mechanisms), indirect tumour toxicity via injury to the microvasculature and a local inflammatory reaction [1].

The photosensitizers (PS) used for thoracic malignancies are classified as non-porphyrins or porphyrins. The latter category includes first, second and third generation PS [3].

Porfimer sodium (Photofrin), a first generation hematoporphyrin PS, has been approved by the U.S. Food and Drug Administration and is the most commonly-used PS in patients with thoracic malignancies [4]. Photofrin offers a variety of advantages over other PS used to treat thoracic malignancies. These include, but are not limited to, the absence of systemic toxicity due to selective drug binding to tumour tissue, the ability to use relatively small doses of the drug, good tissue clearance and the absence of serious side effects due to ongoing use. The most serious side effect is cutaneous photosensitization, which can be prevented by adequate patient education [5].

Meso-tetra-hydroxyphenyl-chlorine, named Temoporfin or Foscan, is another potent second generation PS. It has a short half-life and a hydrophobic nature; these features allow it to be highly photoactivated at 652 nm with preferential accumulation in tumor cells [6,7,8,9]. In addition to direct damage to tumour cells, its pharmacokinetic properties are known to cause profound and persistent vascular damage [10,11]. Talaporfin sodium (TS) ((+)-tetrasodium (2*S*,*S*)-18-carboxylato-20-[*N*-(*S*)-1,2-dicarboxylatoethyl]-carbamoylmethyl-13-ethyl-3,7,1,17-tetramethyl-8-vinylchlorin-2-propanoate) is another second-generation hydrophilic PS with a short plasma half-life [12]. TS activation is known to induce a body-wide immuno-modulation response directed at the tumour and mediated by CD8+ T cells that may help in overcoming tumor resistance [12,13].

In addition to killing of tumour cells, PDT agents can also have a direct impact on tumour vasculature. MV6401 (pyropheophorbide derivative), a second generation PS, has been shown to cause a biphasic response in the vasculature, which includes constriction of the vessels followed by necrosis, thereby delaying tumour growth and proliferation [14].

This review will focus on the use of PDT in non-gastrointestinal, thoracic malignancies, which can be separated into use in parenchymal-based tumours, such as non-small cell lung cancer (NSCLC), and pleural-based tumours, such as mesothelioma and pleural metastases.

## 2. Non-Small Cell Lung Cancer

The use of PDT in NSCLC is typically classified into its use in early stage, otherwise operable cancers or late stage cancers.

### 2.1. Early Cancers

Typically, the standard of care for early stage NSCLC treatment is surgical resection. Thus, the majority of the evidence regarding PDT use in early stage NSCLC exists in patients that are poor surgical candidates.

#### 2.1.2. Radiographically Occult Lung Cancer

**Figure 1:**
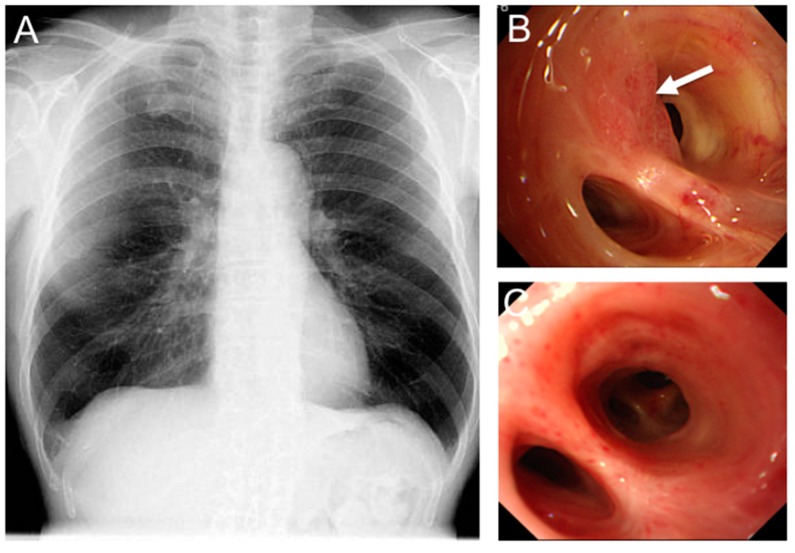
Photodynamic therapy (PDT) for radiographically occult early stage lung cancer. (**A**) A 65-year-old male presented with abnormal sputum cytology and normal chest X-ray; (**B**) Bronchoscopy revealed an endobronchial abnormality at the orifice of the left upper division anterior segmental bronchus (arrow). Biopsy was positive for squamous cell carcinoma; and (**C**) Bronchoscopy six months post PDT (120 J/cm^2^) shows complete response with no residual tumour.

This subset of lung cancers are radiographically occult and are typically discovered via bronchoscopy or sputum cytology (Figure 1) [4,15,16,17].

These patients typically present with central tumours that most often are of squamous cell origin [4,15,16,17]. The use of PDT in these patients has been described mainly in case series or cohort studies, most of which were of a retrospective design. These studies have been mostly small studies with sample sizes ranging from 21 up to approximately 100 patients [15,17,18,19,20,21,22,23]. Complete clinical response rates to PDT have been reported to range from 76%–100% in radiographically occult lung Cancer (ROLC) of sizes ≤1 cm and from 38%–86% in ROLC of sizes >1 cm [17,19,24,25]. Imamura *et al.* reported that the ROLC subgroup with a surface area ≤3 cm^2^ showed a clinical complete response in 72% of patients as compared to a clinical complete response of 64% when all ROLCs were combined [19]. Reported survival rates vary depending on several factors, but most of the studies report five-year overall rates ranging from 43%–72%, with the majority averaging about 50% [18,19,26,27]. The following factors influence the reported survival rates: tumour factors (*i.e.*, size, location), extent of clinical response to therapy, as well as the general medical condition of the patients and their likelihood of experiencing non-cancer mortality. Endo *et al.* reported a group of patients with a remarkable five-year survival of 81%; however, this is likely related to selection bias; this cohort had sub-centimetre lesions and reported a 94% clinical complete response rate [17]. Many studies reported the use of multi-modal therapy, including laser, radiation or surgery in addition to PDT; these were either as part of a planned multi-modal approach or as a salvage strategy in patients who did not demonstrate complete clinical response to PDT [18,19,24,26].

The extent of follow up was not reported in several studies. Where it was reported, the extent of follow up ranged from 4–120 months after treatment and median follow-up periods ranged from 20–78 months [15,17,18,19,21,26,27,28]. Although reporting was not always robust, the data suggest that there is approximately a 25%–30% chance of recurrence at two years [18,19]. However, it appeared that the majority of recurrences were amenable to treatment with repeat PDT, radiation, electrocautery, laser or surgery [18,19,29].

#### 2.2.2. Early Non-Radiographically Occult Lung Cancer (ROLC) Cancers

The use of PDT in early lung cancers has otherwise focused on T1N0M0 cancers or carcinoma *in situ* [18,24,28,29,30,31,32,33,34]. These studies have been mostly small retrospective studies with sample sizes ranging from 13 up to 240 patients [18,24,28,29,30,31,32,33,34]. Complete clinical response rates to PDT have been reported to range from 35%–100% [18,23,24,28,29,30,31,32,33,34,35]. Nearly 100% of tumours achieve some degree of clinical response [28,33,34]. Certain tumour factors are associated with achieving complete clinical response. Tumour size is an important determinant of complete response, with tumours ≤1 cm and >1 cm in size reported to have complete clinical response rates of 94%–98% and 43%–54%, respectively [24,34]. One study further stratified tumours >1 cm in size and reported that clinical complete response rates were 54% and 38% for tumours between 1 and 2 cm and tumours >2 cm in size, respectively [34]. One group, however, has reported that early lung cancers up to 2 cm in size can achieve virtually 100% complete clinical response with PDT [23]. Surface area appears to also be important. Cortese *et al.* reported that the early lung cancer subgroup with a surface area ≤3 cm^2^ showed a clinical complete response rate of 48% as compared to a clinical complete response rate of 0% early lung cancers with surface area >3 cm^2^ [18]. Tumours with clearly visible distal margins on bronchoscopy were also more likely to achieve clinical complete response (87%) as compared to those tumours that did not have visible distal margins (71%) [34].

Reported survival rates vary depending on several factors, but most of the studies report five-year overall rates ranging from 50%–69%, with the majority averaging about 60% [23,24,35,36,37]. Factors influencing reported survival rates include tumour factors (*i.e.*, size, location), extent of clinical response to therapy and the general medical condition of the patients and likelihood of experiencing non-cancer mortality [23,24,35,36]. However, one of the largest studies in this area has shown that although size significantly influences complete clinical response rate, it did not significantly influence overall survival; Furukawa *et al.* showed that although complete clinical response was higher in tumours <1 cm in size (93% *vs.* 58%, *p* < 0.001), five-year overall survival was not (58% *vs.* 59%, *p* = 0.21) [36]. The importance of the general medical condition of these patients is highlighted by the finding in two larger studies that cancer-specific survival was 94% and 93%, whereas overall survival 68% and 69% [23,37]. Many studies reported the use of multi-modal therapy, including laser, radiation (external beam or brachytherapy) or surgery in addition to PDT; these were either as part of a planned multi-modal approach or as a salvage strategy in patients who did not demonstrate complete clinical response to PDT [18,19,23,24,26,28,36,37].

The extent of follow up was not reported in several studies. Where it was reported, the extent of follow up ranged from 2–120 months after treatment, and median follow-up periods ranged from 24–32 months [15,17,18,19,21,23,24,26,27,28,32,36,38].

The quality of reporting of complications is also variable. Moghissi *et al.* performed a review of 626 patients with early central NSCLC treated with PDT from 15 studies and found that the most commonly-reported complications were photosensitivity reactions [35]. They found that the rate of mild sunburn reaction ranged from 5%–28% [35]. They also reported mild respiratory complications (mostly cough and dyspnea) in 0%–18% of patients, as well as mild hemoptysis in 0%–8% of patients [35]. Although serious respiratory complications are possible, these are very uncommon [35,39]. These are usually respiratory failure requiring mechanical ventilation and usually related to airway obstruction due to sloughing off of the necrotic tumour after PDT therapy [19,35,39]. Thus, this highlights the importance of performing bronchoscopic toileting in the days following PDT therapy [39].

### 2.2. Advanced Lung Cancer

PDT has also been described in more advanced stage cancer, most often in the setting of obstructing and often inoperable tumours. The purpose for the use of PDT in this setting is palliation of symptoms. Two randomized trials have been reported in this area. Lam *et al.* compared PDT and external beam radiotherapy to external beam radiotherapy alone in patients with obstructing and inoperable tumours [40]. They reported that 70% (*n* = 14/20) of the PDT group achieved complete clearance of the airway obstruction with no visible tumour as compared to only 9.5% (*n* = 2/21) who achieved the same in the radiotherapy group [40]. Furthermore, the PDT group had significantly lower local recurrence rates [40]. Wieman *et al.* performed a randomized trial in a similar population of patients and compared PDT (*n* = 102) to Nd:YAG laser therapy (*n* = 109) [39]. In an unpublished study, they reported a significantly superior clinical response rate one month after PDT (55%) as compared to laser (30%) [39]. Furukawa *et al.* performed a similar comparison in a non-randomized study of 81 patients treated with PDT and 177 patients treated with Nd:YAG laser [41]. They found that laser had higher rates of clearing endobronchial obstruction (81% *vs.* 75%). This effect seemed to be mainly driven by the superiority of laser in the main bronchi or trachea; in fact, there was no difference in clearing endobronchial obstruction (76% *vs.* 73%) between the two modalities in more distal airways [41]. However, PDT was much better tolerated than laser therapy; no complications reported in the PDT group as compared to the 2% mortality, 3% perforation and 6% massive hemoptysis rates reported in the laser group [41]. Other studies have reported no differences in effectiveness or complications [2,42]. Interestingly, one randomized study suggests that PDT therapy may allow for longer-lasting effects of symptom relief (*i.e.*, from endobronchial obstruction) than laser therapy [2].

In addition to these comparative studies, several prospective case series exist with sample sizes ranging from 10–100 patients; they reported clinical response rates ranging from 41%–100% [43,44,45,46]. The most common adverse events were mild sunburns or light reactions [43,44,45,46]. However, some older series report more serious complications; for example, Vincent *et al.* reported that almost 50% (*n* = 8/17) required intensive care admission, with one patient suffering asphyxiation [46]. Patients prone to experiencing such complications are likely the higher risk patients that have critical endobronchial obstruction; however, outcomes such as this are worrisome in a palliative procedure. In addition to respiratory failure, significant hemoptysis can be a complication and can be fatal [40]. Lam *et al.* reported three deaths due hemoptysis; however, it is important to note that these events occurred at 67, 187 and 567 days after PDT therapy; thus, these remote events were likelier to have been caused by the pre-existing obstructing endobronchial tumour rather than the PDT therapy [40]. Although reporting of symptom control and quality of life was poor, PDT appeared to significantly reduce hemoptysis, dyspnea and coughing as compared to baseline. Due to the poor quality of reporting, it is unclear how clinically significant these reductions were [43,44,45,46].

Table 1 shows a summary of primary studies on PDT in non-small cell lung cancer.

**Table 1:** Summary of primary studies on PDT in non-small cell lung cancer.

Author, Publication Year	Study Type, *n*	Effect of Intervention	Median Follow-up Period (Range)	Complications
*ROLC*				
Noordegraaf, 2003 [15]	Case series, 32 (5 patients received BT + PDT)	5 y OS: 50%	5.3 years (2–11)	Local recurrence, pulmonary fibrosis, emphysema, metastasis, death
Endo, 2009 [17]	Case series, 48	5 y OS: 81%; 10 y OS: 71%	5.25 years (1–12)	Local recurrence, second primary lung cancer, death
Cortese, 1997 [18]	Case series, 21	5 y OS: 72%	5.7 years (2–9.7)	Local recurrence, second primary lung cancer, death
Imamura, 1994 [19]	Case series, 29	5 y OS: 56%	4 years (0.4–6.3)	Local recurrence, pyothorax, pulmonary hypertension, respiratory insufficiency, second primary lung cancer, death
Kato, 1993 [20]	Case series, 58	CR: 82.8%	Not reported	Skin photosensitivity
Furuse, 1993 [24]	Phase II study, 54	CR: 85%	Not reported	Elevation of ALT, pulmonary toxicity, allergic reaction, sunburn
Ono, 1992 [26]	Case series, 36	5 y OS: 43.4%	3.75 years (1–6)	Acute leukemia, photosensitivity, excessive airway secretion, local recurrence
Kato, 2003 [27]	Phase II study, 41	CR: 83%; OS not reported	Not reported	Increased CRP and sputum, cough, fever, neutropenia, leukocytosis, photosensitivity
Patelli, 1999 [28]	Case series, 23	CR: 62%	Not reported	Photosensitivity
Edell, 1992 [29]	Case series, 13	CR: 71%	1.8 years (0.6–4.1)	Increased blood-tinged sputum, sunburns
*Non-ROLC*				
Balchum, 1984 [30]	Case series, 22	CR: 91%	Not reported	Not reported
Li, 1984 [31]	Case series, 21	CR: 12.5%	At least 0.3 years	No major complications reported
Miyazu, 2002 [32]	Case series, 12	CR: 75%	2.3 years (1–3.5)	Not reported
Kato, 1997 [33]	Case series, 26	CR: 82.6%	Not reported	Mild skin photosensitivity
Kato, 1996 [34]	Case series, 240	CR: 39.6%	Not reported	No major complications
Furukawa, 2005 [36]	Case series, 93	5 y OS: 57.9%	0.1–5 years	Not reported
Mccaughan, 1986 [37]	Case series, 18	CR: 40%	Not reported	Local recurrence, second primary lung cancer
Kato, 1997 [38]	Case series, 95	CR: 81%	0.2–16.3 years	Photosensitization
Lam, 1987 [40]	RCT, 11	CR: 40%	0.3–1 years	Excess sputum, hemoptysis, dysphagia, nausea, pruritus, hypercalcemia
Furukawa, 1999 [41]	Case series, 78	CR: 75%	Not reported	Pneumonia, fever, skin sensitivity
Santos, 2004 [42]	Case series, 75	3 y OS: 50%	0.6–1.5 years	No major complications
Diaz-Jiménez, 1999 [2]	RCT, 31	CR: 7%	Not reported	Bronchitis, photosensitization, cough, death
LoCicero, 1990 [43]	Case series, 10	1 y OS: 30%	Not reported	Sunburn, mild anasarca
Moghissi, 1999 [44]	Case series, 100	2 y OS: 19%	1–6 years	Redness of face, mild edema, sunburn
Hugh-Jones, 1987 [45]	Case series, 15	CR: 30%	Not reported	Infection, breathing obstruction
Vincent, 1984 [46]	Case series, 17	CR: 12%	Not reported	Excess secretions, fever, pneumonia, endotracheal candidiasis

Abbreviations: *n* = sample size; y = years; PDT = photodynamic therapy; NSCLC = non-small cell lung cancer; ROLC = radiographically occult lung cancer; BT = bronchoscopic treatment; OS = overall survival; CR = complete response; CRP = C-reactive protein; RCT = randomized controlled trial.

### 2.3. Miscellaneous Used in Non-Small Cell Lung Cancer (NSCLC)

#### 2.3.1. Photodynamic Therapy (PDT) in Downsizing to Allow Resection

PDT has been used as a means of downsizing tumours to allow for resection, as well as reducing the extent of resection necessary [47,48,49]. Konaka *et al.* reported the use of preoperative PDT in 19 patients that allowed them to perform a lobectomy; in these patients, pneumonectomy was thought to have been necessary prior to use of PDT [47]. The denominator in this study is unclear, as they do not report the total number of patients on whom they used preoperative PDT for this indication and how many patients were not spared pneumonectomy [47]. Okunaka *et al.* reported they were successful in downsizing tumours to allow for resection or reducing the extent of resection necessary in 85% (*n* = 22/26) of patients [48]. PDT was performed between two and nine weeks before surgery [48]. Ross *et al.* reported a series of 41 patients in whom pre-operative PDT and chemoradiation were able to downsize tumours to allow for resection or reducing the extent of resection necessary [49]. They reported that 42% (*n* = 10/24) deemed unresectable were able to undergo pneumonectomy, and 27% (*n* = 4/15) of patients thought to require pneumonectomy were downstaged enough to undergo lobectomy [49]. Most interestingly, Ross *et al.* reported that 18% of resected patients had pathologic complete response. It is difficult to assess how much of the complete response was due to the PDT or to the chemotherapy/radiation [49]. However, Ross *et al.* suggest that the majority of the induction response was due to the PDT for the following reasons: (1) gross tumour reduction effects were visible on bronchoscopy 48 hours after PDT therapy; and (2) this gross effect was unlikely to have been as a result of chemoradiation so early in the treatment course [49]. Although there were no deaths at 30 and 90 days after surgery, there were four bronchopleural fistulae after pneumonectomy, three of which were in patients with right pneumonectomy [49].

**Figure 2:**
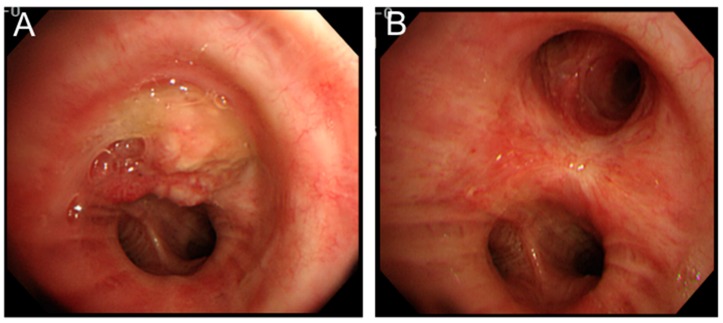
PDT for central type lung cancer. (**A**) Endobronchial tumour (squamous cell carcinoma) completely obstructing the left upper lobe bronchus and extending into the left main stem bronchus; (**B**) complete response to PDT with only scarring on follow up bronchoscopy one year post PDT.

Nakajima *et al.* reported a case wherein PDT alone was sufficient to cure a tumour obstructing the left upper bronchus [50]. They reported that PDT was initially used as a means of downstaging the tumour and facilitating a sleeve left upper lobectomy [50]. However, the tumour had a complete response, and resection was avoided; thus, the patient avoided a lobectomy, had preserved lung function and was recurrence-free at five years after PDT (Figure 2) [50].

#### 2.3.2. Salvage Therapy for Recurrences

**Figure 3:**
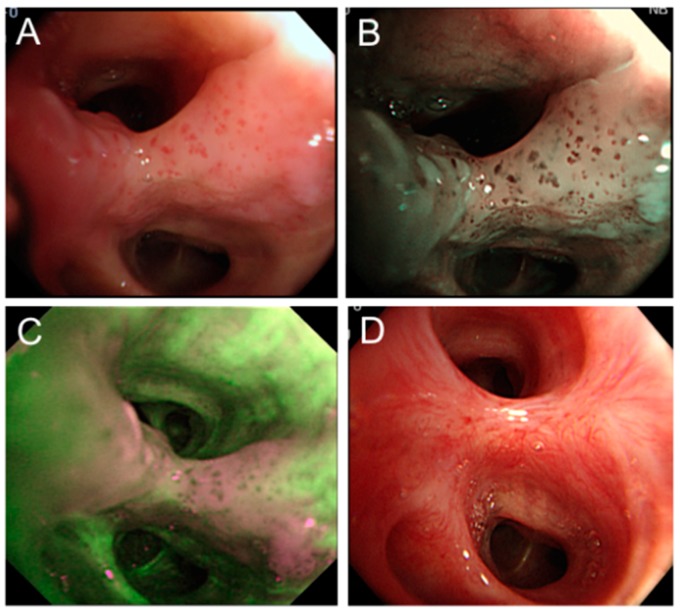
PDT for recurrent lung cancer. (**A**) A 70-year-old male with history of left upper lobectomy for stage IA T1aN0M0 squamous cell carcinoma. Bronchoscopy performed 10 years post resection for the investigation of abnormal sputum cytology revealed abnormal bronchial mucosa in the left lower lobe basal lateral segmental bronchus; (**B**) Narrow band imaging shows dotted vessels within the abnormal mucosa; (**C**) Autofluorescence bronchoscopy shows abnormal fluorescence in the same area; and (**D**) Follow-up bronchoscopy six months post PDT (180 J/cm^2^) shows only scarring from the treatment with complete response.

PDT has been used as a salvage or rescue therapy in patients who have local recurrence after resection or radiation therapy (Figure 3).

Moghissi *et al.* reported this use in 10 patients and found no complications with use of PDT [51]. They also reported that all patients had complete response to PDT [51]. However, they also reported that many patients required repeat PDT in order to treat recurrence within 15 months of the initial PDT application [51]. Corti *et al.* reported 28 patients who had PDT for local recurrence after resection and reported no significant complications [52]. They reported an approximately 73% complete response rate with about one third of patients experiencing local recurrence with 3–108 months after initial PDT therapy; recurrences after PDT therapy were treated with repeat PDT or radiation therapy [52].

### 2.4. Methodologic Issues

The literature in this area involves several methodologic issues that warrant discussion and consideration when attempting to interpret the evidence. First and foremost, the majority of literature is comprised of non-randomized case series; as such, caution should be used when interpreting the results of these mostly non-comparative data. The quality of reporting is suboptimal, but appears mostly reliable as it relates to adverse events; that being said, there is a wide variability in adverse event rates, which can be a cause for concern regarding the accuracy of reporting.

A major issue is the lack of a standard definition of “clinical response”. Since this is often the main outcome of studies in this area, lack of a standard definition is problematic both for interpretation of results of single studies, as well as for evidence synthesis (e.g., in meta-analysis). Another major issue is that PDT is often used in concert with other modalities of therapy, and the combinations are quite variable among all of the studies. Thus, it becomes difficult to tease apart the independent effect of PDT therapy. This seems to be less of a concern in early stage central tumours or ROLCs. In some studies of early stage central tumours or ROLCs, it does appear that stand-alone therapy with PDT is a viable option with the use of alternative therapies as potential salvage for partial response or recurrence. Although the few randomized studies had good follow-up data, this was not the case for the majority of the studies. Reasons for loss to follow up were not reported consistently, and thus, it is not possible to determine the impact of loss to follow up on outcome differences between those who received PDT or other therapies. Loss to follow up has been shown in other areas of medicine to potentially change the conclusions of even higher quality studies [53,54,55]. Thus, going forward, future studies of PDT would ideally consist of comparative studies of PDT comparing the effectiveness of using PDT or adding PDT to other therapies *vs.* not using or adding PDT, rather than being pure case series with no comparison group. These would ideally be randomized trials, but good propensity-matched observational studies would also be informative. Going forward, good documentation and reporting of follow-up data are also important. Finally, it would be ideal to gather a consensus definition of “clinical response” so as to allow for better interpretation of single studies, as well as to facilitate better evidence synthesis.

## 3. Pleural Malignancies

A multitude of studies have investigated the therapeutic benefit of PDT in both primary pleural malignancies, namely malignant pleural mesothelioma (MPM), as well as metastatic disease to the pleura from other thoracic malignancies.

### 3.1. Malignant Pleural Mesothelioma

Intraoperative PDT for treatment of MPM was first investigated by Pass and colleagues at the National Cancer Institute in the late 20th century . In their phase III clinical trial, 63 patients with localized MPM were randomized to multimodal treatment (maximum debulking surgery and postoperative chemotherapy with cisplatin, tamoxifen immunochemotherapy and interferon α-2b) with or without PDT [56]. They concluded that patients with high stage disease are amenable to multimodal therapy and that there is no impact of first-generation PDT on local control of disease or overall survival [56].

Since then, numerous groups have attempted to further elucidate the therapeutic benefits of intraoperative PDT in mesothelioma patients [57,58,59,60,61,62,63,64,65,66]. PDT can be an effective component of a surgery-based multimodal treatment plan that offers improved local control of disease and survival rates.

Recently, Friedberg *et al.* piloted a phase I–II trial, with 38 mesothelioma patients [67]. PDT administration, following a thorough pleurectomy/decortication, yielded a 15-month median disease-free progression and a 42-month median survival time for epithelial histology, a marked increase from previously-quoted survival times (24 months) after combination therapy with chemo-radiation and surgery [68]. Although the reason for this increase in survival time is not clear, it may be potentially related to sparing the lung during surgery and/or a PDT-related phenomenon. The results therefore warrant further investigation. Nonetheless, they offer significant promise for MPM patients and have become the basis for an ongoing phase II trial (NCT NCT02153229), a proposed randomized phase III trial and research efforts into the basic science of PDT (NCT02106559) [69].

One must proceed with caution, however, as dose-related toxicity and complications associated with the procedure may preclude recommendation of PDT for widespread use [70]. Esophageal fistulas, for instance, are a serious side effect of intrathoracic PDT. They are often a delayed complication after PDT presenting two weeks–two months post treatment with cough, lung infiltrates and fever. Surgical management of this complication includes drainage of the remaining esophageal remnants and esophageal bypass. In addition, total parenteral nutrition may be necessary for successful palliation of complications [71].

### 3.2. Pleural Metastases

Metastasis to the pleura from a malignancy in the thorax is challenging to manage clinically due to controversial treatment options. Pleural carcinomatosis patients have a median survival time that ranges from 6–9 months [72,73,74,75,76]. Currently, pleural spread may be managed using a combination of chemotherapy, photodynamic therapy and surgery with pleurectomy [77].

In a phase II trial investigating pleural spread from NSCLC, patients were treated surgically with total resection or debulking of tumor, as well as intraoperative PDT [78]. The trial showed local control of pleural disease at six months in 73.3% of patients and a 21.7-month median survival time. This is significantly longer compared to patients (6–9 months) treated without surgery and PDT [78]. It is important to note that the effectiveness of PDT in comparison to a control group in the same center is unknown [78]. It is imperative to further explore the impact of centre-specific practices on patient mortality and survival.

Chen *et al.* also examined 18 patients with thymoma or lung cancer with metastases to the pleura who underwent pleural PDT and surgery [77]. In comparison to those receiving chemotherapy (*n* = 51), PDT lung cancer patients had better overall survival (mean survival time: 39.0 *vs.* 17.6 months; *p* = 0.047) [77]. Thymoma patients treated with PDT also experienced better local control of disease than their non-PDT counterparts [77]. The authors concluded that with rigorous selection of patients, radical surgical resection with adjunctive PDT for pleural metastases in those with NSCLC or thymoma is practical and provides a benefit towards the overall survival of the patient [77].

Table 2 depicts a summary of primary studies on PDT in pleural malignancies.

**Table 2:** Summary of primary studies on PDT in pleural malignancies.

Author, Publication Year	Study Type, *n*	Effect of Intervention	Median Follow-up Period (Range)	Complications
**Malignant Pleural Mesothelioma**				
Pass, 1997 [56]	Phase III trial, 63	Median survival: 1.2 y	Not reported	Bronchopleural fistula
Du, 2010 [60]	Case series, 11	Not reported	Not reported	Radiation pneumonitis
Friedberg, 2003 [63]	Phase I trial, 26	MTD: 0.1 mg/kg of Foscan PDT	Not reported	Capillary leak syndrome, wound burns, photosensitivity
Moskal, 1998 [64]	Case series, 40	2 y OS: 23%	Not reported	A-fib, sepsis, bronchopleural fistula, empyema
Baas, 1997 [65]	Case series, 5	CR: 80%	8-11 months	Metastasis, skin sensitivity
Friedberg, 2012 [67]	Retrospective cohort, 38	CR: 97%	2.9 years	Stroke, transient respiratory insufficiency, a-fib, chyle leak
Schouwink, 2001 [70]	Phase I/II, 28	CR: 50%, MTD: 0.15 mg/kg	2.6 years (0.8–4.4)	A-fib, diaphragm rupture, empyema, depression, mucus impaction, death
Luketich, 1996 [71]	Case report, 1	Not reported	Not reported	Esophagopleural fistula
**Pleural Metastases**				
Chen, 2015 [77]	Case series, 18	5y OS: 57.4%	3.2 years	Acute respiratory distress syndrome, air leakage, skin redness
Friedberg, 2004 [78]	Phase II, 22	CR: 73.3%; 1 y OS: 68%	2.8 years	Transaminitis, edema, transient thrombocytopenia, fever

Abbreviations: *n* = sample size; y = years; PDT = photodynamic therapy; OS = overall survival; CR = complete response; MTD = maximally-tolerated dose.

## 4. Conclusions

PDT has a role in the management of early and late thoracic malignancies. It can be used facilitate minimally-invasive treatment of early endobronchial tumours and also to palliate obstructive and bleeding effects of advanced endobronchial tumours. PDT has been used as a means of downsizing tumours to allow for resection, as well as reducing the extent of resection necessary. It has also been used successfully for minimally-invasive management of local recurrences, which is especially valuable for patients who are not eligible for radiation therapy. PDT has also shown promising results in mesothelioma and pleural-based metastatic disease. As new generation photosensitizers are being developed and tested and methodological issues continue to be addressed, the role of PDT in thoracic malignancies continues to evolve.

## Abbreviations

PDT: photodynamic therapy
PS: photosensitizer
NSCLC: non-small cell lung cancer
ROLC: radiographically occult lung cancer
MPM: malignant pleural mesothelioma
